# Risk factors for repeat percutaneous coronary intervention in young patients (≤45 years of age) with acute coronary syndrome

**DOI:** 10.7717/peerj.6804

**Published:** 2019-04-26

**Authors:** Tianwen Han, Qun Wang, Huanwan Yang, Shanshan Zhou, Jing Wang, Jing Jing, Tao Zhang, Yuqi Liu, Yundai Chen

**Affiliations:** Chinese PLA General Hospital, Beijing, China

**Keywords:** Risk factor, Young, Revascularization, Acute coronary syndrome

## Abstract

**Background:**

The incidences of premature coronary heart disease present a rising trend worldwide. The possible risk factors that may predict the incidence of repeat percutaneous coronary intervention (PCI) in premature acute coronary syndrome (ACS) remains unclear.

**Methods:**

A total of 203 patients ≤45 years with ACS from Chinese PLA General Hospital who have undergone angiography twice were included in this report. Data were collected from medical records of patients during hospitalization. Baseline characteristics which have significant differences in the univariate analysis were enrolled into the multiple logistic regression analysis. According to the odds ratio (OR) of these variables, different values were assigned to build a risk model to predict the possible risk of the premature ACS patients undergoing repeat PCI.

**Results:**

Of the 203 young patients, 88 patients (43.3%) underwent repeat PCI. The intermit time (OR 1.002, (95% CI [1.001–1.002])), diastolic blood pressure of second procedure (OR 0.967, (95% CI [0.938–0.996])), stent diameter (OR 0.352, (95% CI [0.148–0.840])), HbA1C of the first procedure (OR 1.835, (95% CI [1.358–2.479])), and Troponin T of the second procedure (OR 1.24, (95% CI [0.981–1.489])) were significantly associated with the incidence of repeat PCI in patients with premature ACS. An aggregate score between 0 and 6 was calculated based on these cutpoints.

**Conclusion:**

For young patients with premature ACS, risk of undergoing repeat PCI was high. HbA1C was a significant, independent predictor for the incidence of repeat revascularization, and weighed more than traditional lipid profile. The glucose metabolism and disorders in patients with premature ACS should be routinely screened.

## Introduction

The incidence of coronary artery disease (CAD) is increasing worldwide (Benjamin et al., 2018). According to Chinese Cardiovascular Disease Report, 11 million patients have CAD (Chen et al., 2017). The burden of cardiovascular disease in China is increasing, and is gradually becoming a major public health problem. With changes in lifestyle, dietary habits, and social stress, the onset age of CAD has decreased. Although compared with elderly patients, the prognosis for young patients with CAD may be relatively good (Lautamaki et al., 2014), risk factors and prognosis for premature acute coronary syndrome (ACS) are still debilitating.

According to the VIRGO study, almost 98% of young patients (18–55 years) with acute myocardial infarction have one or more risk factors for CAD and 64% have three or more risk factors; only about half (53%) of patients knew that they had CAD risks, and even fewer were informed about the risk factors for CAD before the onset of the disease (Leifheit-Limson et al., 2015). Besides, some studies have shown that young patients with ACS had a higher plaque burden than older patients (Chaudhary et al., 2017).

Since the pathophysiology of atherosclerosis in young ACS patients is different from older patients by the presence of genetic abnormalities, lipids metabolism, and coronary artery anomalies, the risk factors profile in young ACS patients need to be addressed (Barbero et al., 2017). It is of great significance to identify young individuals who are at risk for cardiovascular events, as such patients may need more aggressive secondary prevention. However, predicting risks in young ACS patients is challenging, and how to identify young ACS patients as high risk for recurrent cardiovascular events is still unclear (Gupta et al., 2013). Repeat revascularization is one of the most common cardiovascular events after ACS. The rate of re-PCI in young ACS patients is unknown. Therefore, it is very important to identify high-risk patients who will likely require a repeat percutaneous coronary intervention (PCI). In this study, we analyzed the risk factors for repeat PCI treatment in young ACS patients.

## Materials and Methods

### Study population

**Figure 1:**
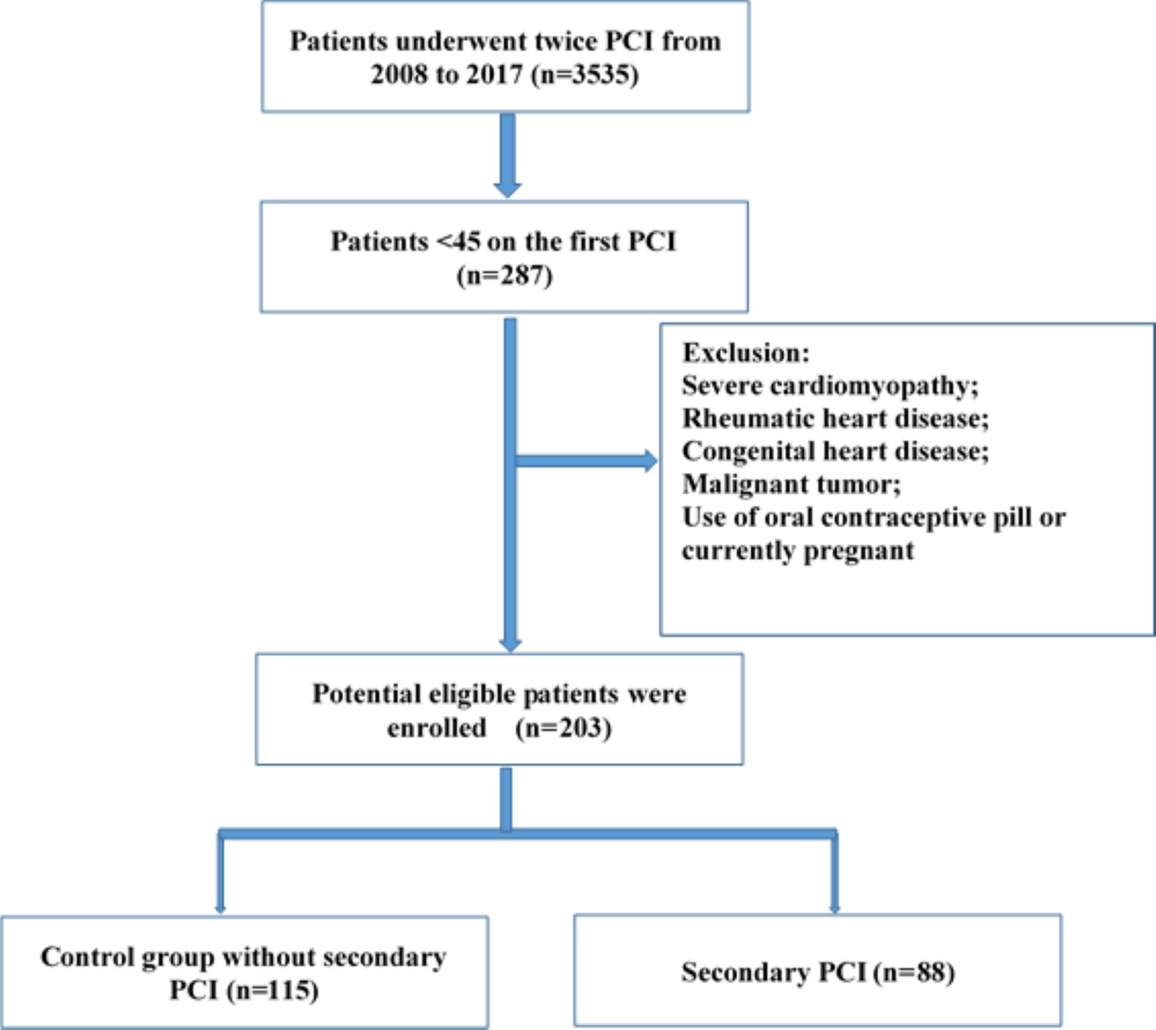
Flow chart of the procedure of enrolling subjects.

All included patients were hospitalized and underwent coronary angiography (CAG) twice at our cardiovascular center from 2008 to 2017. Two hundred eighty-seven patients were younger than 45 years at the time of first PCI treatment. Patients with severe cardiomyopathy, rheumatic heart disease, congenital heart disease, malignant tumors, use of oral contraceptive pills, current pregnancy, those for whom a second PCI was recommended for advanced plaque but who refused this treatment, and those with coronary artery bypass grafting were excluded. Patients were divided into two groups: the control group and re-PCI group. In the control group, repeat CAG showed no need for a second PCI; in the re-PCI group, repeat CAG showed significantly advanced plaque requiring repeat treatment including in-stent restenosis and clinically driven revascularization (Fig. 1). A revascularization considered clinically indicated if angiography at follow-up shows a percent diameter stenosis ≥50% and if one of the following exists: (1) positive history of recurrent angina pectoris, presumably related to the target vessel; (2) objective signs of ischemia or during exercise test, presumably related to the target vessel; (3) A target lesion revascularization or target vessel revascularizaion with a diameter stenosis ≥70%. The study protocol was approved by the Chinese PLA General Hospital Review Boards (NO. S2018-083-02). Informed consent for the procedures and consent for publication were obtained from all participants.

All enrolled patients met the following inclusion criteria: 1) underwent CAG twice, accepted initial PCI treatment, and had a CAG interval longer than 6 months; 2) were less than 45 years of age at initial PCI treatment; and 3) diagnosed with ACS at first hospitalization. Records of all hospitalized patients were collected from the medical database. We compared patient age at initial procedure, interval between CAG procedures, sex ratio, and other parameters between the groups.

### Coronary angiography procedures and imaging evaluation

Before the procedure, all patients were administered 300 mg aspirin and 300 mg clopidogrel or 180 mg ticagrelor. After local disinfection and anesthesia, Seldinger puncture was successfully performed for CAG and interventional therapy. During the procedure, intravenous heparin was administered to keep the activated clotting time between 250 and 300 s. The culprit lesions and stent position, mean diameter, and mean length were compared between the groups.

Coronary angiography images in at least two vertical planes were taken for quantitative CAG analysis. Guiding-catheter calibration was used to obtain CAG images of vessel diameter and other parameters. Reference vessel diameter was defined as the diameter of the relevant vessel segment proximal and distal to the lesion. The minimum diameter of the lesion was defined as the diameter of the coronary artery at the narrowest point of the lesion. Absolute internal diameter loss was equal to the difference in minimum lesion diameter from immediately postoperatively to follow-up. Relative diameter loss was defined as the ratio of the absolute inner diameter loss to the reference vessel diameter. The success criteria for PCI were residual stenosis <20%, with no large branch occlusion or severe dissection. Restenosis was defined as vascular stenosis ≥50% at the lesion site during follow-up.

### Statistics analyses

Measurement data were compared with an independent *t*-test and expressed as mean ± standard deviation; count data were compared with a chi-square test and expressed as percentages. *p* < 0.05 was defined as indicating a significant difference. Statistical analysis was performed with the SPSS 18.0 software package. The raw data applied for analyses are contained in Dataset S1. To identify potential risk factors, we chose variables with *p* < 0.1 for logistic regression analysis. Variables with *p* < 0.05 were significantly different and were included in the model. We assigned values to each of the included variables on the basis of the odds ratio (OR) value. We further compared the risk scores of the control group and the re-PCI group. Risk stratification was performed according to the model and was divided into low-risk, intermediate-risk, and high-risk categories.

## Results

### Baseline characteristics

Baseline characteristics were compared between subjects with and without re-PCI (Table 1). Compared with the control group, the re-PCI group have higher level of HbA1C (7.28 ± 1.21 vs. 6.50 ± 1.14, *p* = 0.000), more culprit lesions in left anterior descending artery (65.9% vs. 51.3%, *p* = 0.036), and smaller stent diameter (3.03 ± 0.39 vs. 3.16 ± 0.39, *p* = 0.023) at the first CAG. And in the follow-up CAG (Table 2), the re-PCI group were older (41.3 ± 4.5 vs. 43.1 ± 4.4, *p* = 0.007), had higher level of fasting glucose (6.35 ± 2.87 vs. 7.33 ± 3.31, *p* = 0.025) and troponin T (0.01 ± 0.04 vs. 0.09 ± 0.29, *p* = 0.004), other variables were comparable between two groups.

**Table 1:** Baseline characteristics of first PCI.

	Control (*N* = 115)	re-PCI (*N* = 88)	*p* value
Age, y	39.9 ± 4.4	40.7 ± 3.9	0.171
Male, *n* (%)	108 (93.9)	80 (90.9)	0.417
BMI, kg/m^2^	27.6 ± 4.2	28.0 ± 3.8	0.495
SBP, mmHg	126.3 ± 16.9	125.8 ± 15.3	0.852
DBP, mmHg	77.6 ± 11.7	76.0 ± 11.6	0.776
HR, b/m	78.8 ± 15.4	76.1 ± 13.1	0.196
Hypertension, *n* (%)	57 (49.6)	48 (54.4)	0.481
DM, *n* (%)	27 (23.4)	28 (31.8)	0.185
Current smoker, *n* (%)	82 (71.3)	60 (68.2)	0.631
Previous MI, *n* (%)	16 (13.9)	12 (13.6)	0.954
Glucose, mmol/l	7.14 ± 2.97	7.35 ± 3.45	0.644
HbA1C, mmol/l	6.50 ± 1.14	7.28 ± 1.21	0.000
TC, mmol/l	4.20 ± 0.95	4.32 ± 1.25	0.468
Triglycerides, mmol/l	1.98 ± 1.09	1.97 ± 1.32	0.984
LDL, mmol/l	2.61 ± 0.87	2.65 ± 1.06	0.739
HDL, mmol/l	0.92 ± 0.23	0.92 ± 0.24	0.947
CKMB, ng/ml	40.1 ± 101.4	45.7 ± 97.9	0.691
Troponin T, ng/l	1.49 ± 4.33	2.15 ± 5.71	0.35
Presentation, *n* (%)			
NSTEMI	19 (16.5)	19 (21.5)	0.358
STEMI	27 (23.5)	17 (19.3)	0.475
Culprit lesion, *n* (%)			
LAD	59 (51.3)	58 (65.9)	0.036
LCx	19 (16.5)	7 (7.9)	0.07
RCA	33 (28.7)	21 (18.3)	0.44
LM	4 (3.5)	2 (2.2)	0.932
Stent position, *n* (%)			
LM	7 (6)	2 (2.2)	0.334
LAD	75 (65.2)	64 (72.7)	0.253
LCx	28 (24.3)	22 (25)	0.914
RCA	45 (39.1)	31 (35.2)	0.701
Stent length, mm	23.9 ± 5.9	24.6 ± 5.3	0.37
Stent diameter, mm	3.16 ± 0.39	3.03 ± 0.39	0.023
Bifurcation stents, *n* (%)	2 (1.7)	2 (2.2)	0.786
Slow/no flow, *n* (%)	2 (1.7)	1 (1.1)	0.724
Medication during hospitalization, *n* (%)			
Aspirin	114 (99.1)	88 (100)	–
Clopidogrel	111 (96.5)	87 (98.8)	0.541
Ticagrelor	18 (15.6)	13 (14.8)	0.862
DAPT	114 (99.1)	88 (100)	–
Statin	110 (95.7)	85 (96.6)	0.981

**Note:**

BMI, body mass index; SBP, systolic blood pressure; DBP, diastolic blood pressure; HR, heart rate; DM, diabetes mellitus; TC, total cholesterol; TG, Triglycerides; LDL-C, low-density lipoprotein cholesterol; HDL-C, high-density lipoprotein cholesterol; STEMI, ST-elevated myocardial infarction; NSTEMI, non-ST elevated myocardial infarction; LM, left main artery; LAD, left anterior descending branch; LCX, left circumflex artery; RA, right artery; DAPT, dual antiplatelet therapy.

**Table 2:** Characteristics of second angiography.

	Control (*N* = 115)	re-PCI (*N* = 88)	*p* value
Age, y	41.3 ± 4.5(y)	43.1 ± 4.4(y)	0.007
Interval time, d	513.0 ± 349.8	844.6 ± 567.0	0.000
BMI, kg/m^2^	27.1 ± 3.3	27.4 ± 4.4	0.646
SBP, mmHg	128.8 ± 15.1	125.4 ± 15.6	0.116
DBP, mmHg	78.7 ± 11.4	77.4 ± 11.5	0.1
HR, b/m	75.1 ± 11.7	74.6 ± 11.9	0.788
Hypertension, *n* (%)	62	52	0.461
DM, *n* (%)	30	31	0.461
Current smoker, *n* (%)	81	61	0.789
Previous MI, *n* (%)	52	39	0.898
Glucose, mmol/l	6.35 ± 2.87	7.33 ± 3.31	0.025
HbA1C, mmol/l	6.50 ± 0.98	6.71 ± 1.25	0.204
Total cholesterol, mmol/l	3.50 ± 0.83	3.52 ± 1.08	0.85
Triglycerides, mmol/l	1.58 ± 0.82	1.71 ± 0.755	0.263
LDL, mmol/l	2.08 ± 0.71	2.09 ± 0.91	0.951
HDL, mmol/l	0.92 ± 0.21	0.89 ± 0.23	0.366
CKMB, ng/ml	4.2 ± 5.1	5.8 ± 13.6	0.228
Troponin T, ng/l	0.01 ± 0.04	0.09 ± 0.29	0.004
Presentation, *n* (%)			
NSTEMI	2	5	0.255
STEMI	0	4	–
Stent length, mm	–	23.0 ± 7.1	–
Stent diameter, mm	–	3.02 ± 0.46	–
Aspirin	112	88	–
Clopidogrel	107	79	0.405
Ticagrelor	16	18	0.216
DAPT	112	88	–
Statin	112	86	0.761

**Note:**

BMI, body mass index; SBP, systolic blood pressure; DBP, diastolic blood pressure; HR, heart rate; DM, diabetes mellitus; TC, total cholesterol; TG, Triglycerides; LDL-C, low-density lipoprotein cholesterol; HDL-C, high-density lipoprotein cholesterol; STEMI, ST-elevated myocardial infarction; NSTEMI, non-ST elevated myocardial infarction; LM, left main artery; LAD, left anterior descending branch; LCX, left circumflex artery; RA, right artery; DAPT, dual antiplatelet therapy.

### Predictors of re-PCI in young ACS patients

Logistic regression analysis with all included variables showed that the time interval between CAG procedures (OR 1.002, (95% CI [1.001–1.002])), diastolic blood pressure (DBP) at the second CAG (OR 0.967, (95% CI [0.938–0.996])), stent diameter (OR 0.352, (95% CI [0.148–0.840])], HbA1C at the first CAG (OR 1.835, (95% CI [1.358–2.479])), and TnT at the second CAG (OR 1.241, (95% CI [0.981–1.489])) were significantly different between groups. These variables were included in the risk model (Table 3).

**Table 3:** Multiple logistic regression analysis.

	B	S.E.	Wals	*p*	HR (95% CI)
Intermit time	0.001	0	18.858	0	1.002 [1.001–1.002]
DBP	−0.034	0.015	5.092	0.024	0.967 [0.938–0.996]
Stent diameter	−1.004	0.444	5.53	0.019	0.352 [0.148–0.840]
HbA1C	0.607	0.153	15.634	0	1.835 [1.358–2.479]
TnT	2.387	4.787	7.323	0.007	1.241 [0.981–1.489]

**Note:**

DBP, diastolic blood pressure; HbA1C, Hemoglobin A1c; TnT, Troponin T.

### Risk model stratification

**Figure 2:**
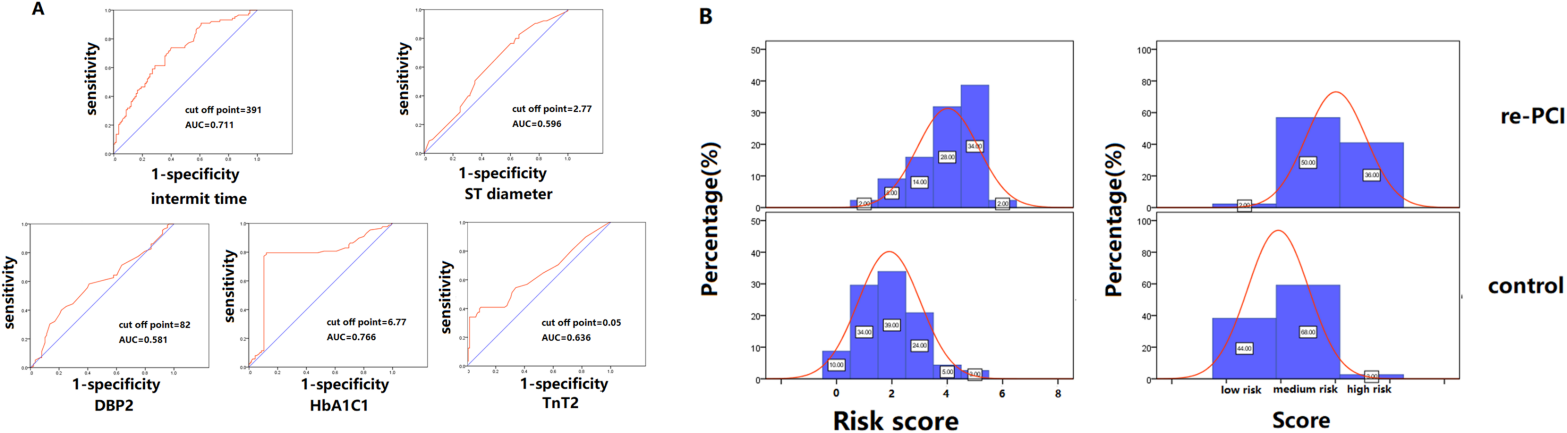
Risk model stratification and the distribution frequency of different risk score. (A) The cutoff points of the variables, including interval time, DBP at the second CAG, stent diameter, HbA1C at the first CAG and TnT at the second CAG, were analyzed with receiver operating characteristic curves to find the maximum area under the curve. (B) The comparison of the frequency distribution of re-PCI and control groups. The high-risk frequency of the second PCI group was significantly higher than that of the control group.

**Figure 3:**
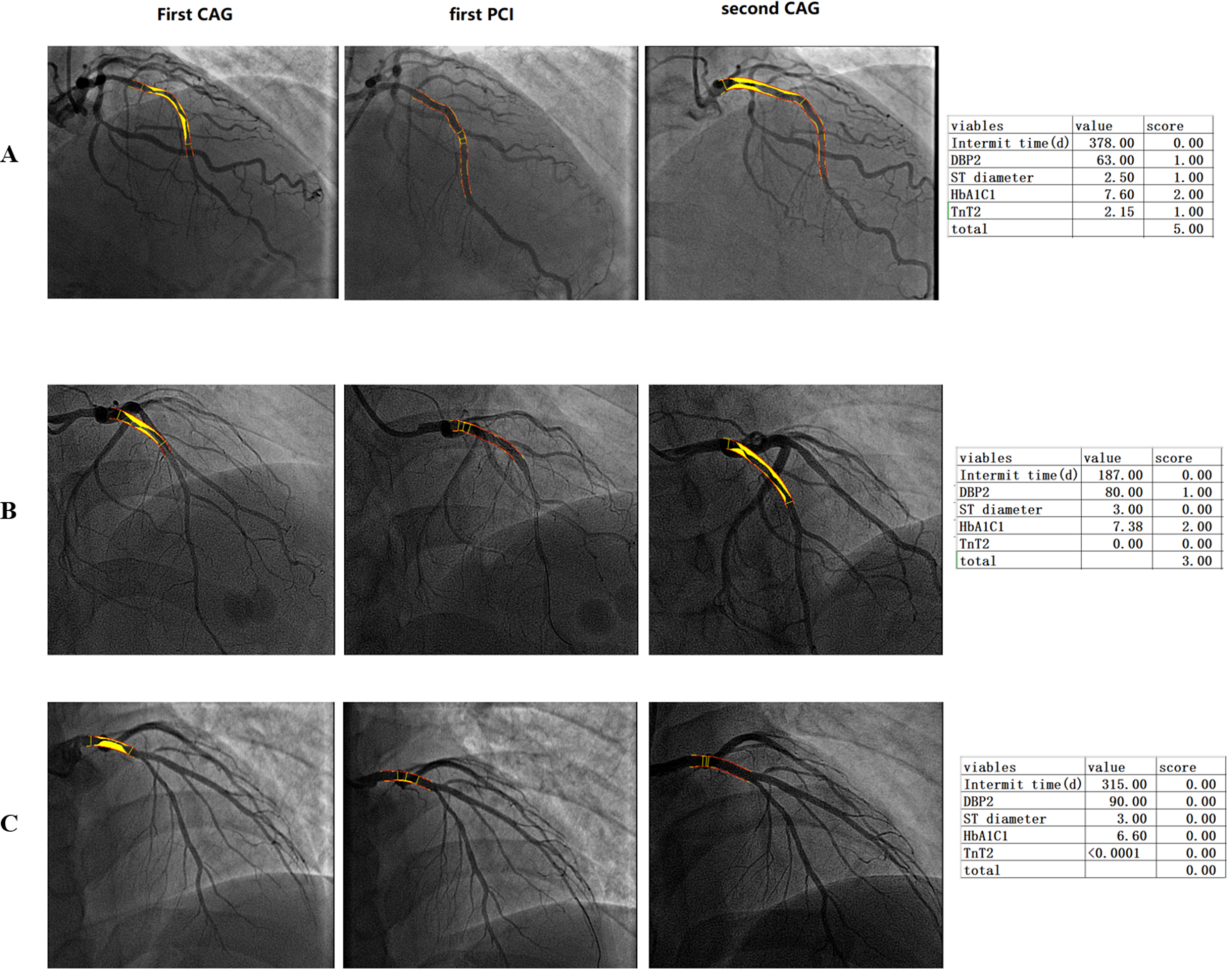
Imaging of patients with low, middle and high-risk stratification who underwent repeat percutaneous coronary intervention. (A) High-risk score group, a 33-year-old patient with a high score, HbA1C of 7.36, and total occlusion of the proximal LAD. Repeat CAG showed total occlusion of the proximal stent. (B) Middle-risk score group, a 44-year-old patient with a medium risk score and an HbA1C of 7.38 who had one stent implanted in the proximal LAD. Repeat CAG showed in-stent restenosis of the proximal segment of the LAD. (C) Low-risk score group, a patient with a total score of 0 experienced no plaque progression.

Variables were assigned to the risk model according to their OR. The cutoff points of these variables were analyzed with receiver operating characteristic curves to find the maximum area under the curve (Fig. 2A). CAG procedure interval time longer than 391 days was assigned 1 point. DBP ≤82 mmHg at the second CAG procedure was assigned 1 point. Stent diameter ≤2.77 mm was assigned 1 point. HbA1C ≥6.77 at the first CAG was assigned 2 points. TnT ≥0.05 at the second procedure was assigned 1 point. The frequency of different scores according to group was shown in Fig. 2B. The average score of the re-PCI group was higher than that of the control group (3.0 ± 1.3 vs. 2.4 ± 1.2, *p* < 0.001). According to the distribution frequency, a score of 0–2 indicated low risk of repeat PCI, 3–4 indicated medium risk, and 5–6 indicated high risk (Table 4). The examples of risk stratification were shown in Fig. 3.

**Table 4:** Risk-prediction model.

	Score
Intermit time	
<391 days	0
≥391 days	1
DBP	
>82 mmHg	0
≤82 mmHg	1
Stent diameter	
>2.77 mm	0
≤2.77 mm	1
HbA1C	
<6.77	0
≥6.77	2
TnT	
<0.05	0
≥0.05	1

**Note:**

DBP, diastolic blood pressure; HbA1C, Hemoglobin A1c; TnT, Troponin T.

## Discussion

Although several studies have described the risk factors associated with the need for repeat revascularization after PCI, few have reported the incidence and predictors of repeat PCI in young patients. Our study demonstrated that for young patients with CAD, the most significant risk factors for repeat PCI were baseline HbA1C, stent diameter, and DBP and TnT at the second CAG procedure. This study supports the findings of prior studies that have demonstrated robust associations between premature CAD, glucose metabolism, and myocardial injury. The cardiac–metabolic metrics warrant more investigation in young CAD patients.

Our previous study demonstrated a rising trend in the incidence of CAD among young people in China from 2010 to 2014, especially among men (Wang et al., 2017). This trend may be partially attributed to unhealthy lifestyle and uncontrolled risk factors such as diabetes, hypertension, and obesity. Singh et al. (2018b) reported that the prevalence of ST-segment elevation myocardial infarction among young individuals was 12.8%, and that among 200,000 PCI performed annually in women in the US, 21% were in patients <50 years of age, which places great burdens on society and families (Dehmer et al., 2012). A recent study in New Zealand reported that one-quarter of patients with a first-time cardiovascular disease event were aged <55 years; younger patients had a very high risk-factor burden, with more smoking, higher body mass index, and higher prevalence of diabetes than older patients (Earle et al., 2018). Barbero et al. (2017) found that younger patients with ACS were more likely to have plaque rupture than older patients and that their plaques were characterized by less fibrotic and calcific components, which creates vulnerable plaque. This finding may explain why premature CAD may be less prevalent than CAD in older patients but has worse outcomes. Despite the high risk and incidence of young ACS, the prevention and treatment for young patients seem to be underestimated. In YOUNG-MI registry, it is reported that at least one cardiovascular risk factor was present in 83% patients while 63% women and 46% men were not eligible for current guideline-based treatment thresholds prior to their MI. These findings highlight a great need for better risk assessment among young adults (Singh et al., 2018a). Furthermore, for those young patients who have undergone ACS, few studies have evaluated their risk factors and the current treatment has no difference between young and older patients.

In this study, we first evaluated the risk factors that predict the need for repeat revascularization in patients aged less than 45 years. It is well established that diabetes mellitus is an independent predictor of adverse cardiovascular events, regardless of age (Kim et al., 2013; Lin et al., 2017). However, despite the traditional paradigm, traditional risk factors could not completely explain the patterns of repeat revascularization in young CAD patients; whether these factors can predict prognosis also remains unknown. In this study, we did not find differences in lipid profiles between the repeat PCI group and the control group. This finding suggests that under optimized medical therapy, traditional lipid metrics play a limited role in predicting outcomes in young patients. However, it is worth mentioning that the average LDL-C level was not under ideal control in patients in this study, which needs more attention (Manocha et al., 2014). In a recent study, Chieng et al. (2018) reported that elevated lipoprotein (a) and LDL-C were significant predictors of CAD severity and complexity in patients with premature CAD. However, our study did not reveal a difference in lipoprotein (a) or LDL-C between groups, perhaps because we focused on young patients who had undergone stent implantation and medical therapy to modify lipid levels. Furthermore, we found a large difference between groups in both HbA1c and fasting glucose, which indicates that glucose control warrants more attention in young patients with higher HbA1c. Consistent with prior studies, the prevalence of diabetes mellitus presents as a great burden for young patients. In the ARIC Community Surveillance Study, the history of diabetes mellitus has increased from 25% to 35% among young MI patients during the past 20 years (Arora et al., 2019). Patients with diabetes are known to be at higher risk of cardiovascular disease and to have worse outcomes (Sarwar et al., 2010; Selvin et al., 2004). Our results are consistent with prior studies. Lin et al. (2017) reported that patients with diabetes mellitus have higher mortality after PCI than patients with hypertension. Earle et al. (2018) reported that young CAD patients <50 years of age in New Zealand had higher median HbA1c than older patients, and that their diabetic control was significantly worse, according to HbA1C levels. In the present study, we found that HbA1C was an independent risk factor predicting repeat PCI, regardless of the presence of diabetes; glucose control needs to be monitored and further modified in these young patients.

Our study found that young patients with higher TnT were more likely to undergo repeat PCI than patients with lower TnT. Cardiac troponin concentration is a marker of myocardial injury; its elevation is strongly associated with an adverse prognosis in patients with ACS (Bhatt et al., 2004; Morrow et al., 2001). Thus it is reasonable that a higher TnT level, which indicates myocardial injury, would be associated with the need for repeat revascularization in young patients with premature CAD. One study showed that patients with both type 2 diabetes and stable ischemic heart disease who had a higher TnT level at 1-year follow-up had worse outcomes than patients with lower TnT levels (Everett et al., 2015; Keller et al., 2018). In our study, the relationship between TnT and the risk of repeat revascularization suggests that TnT concentration is a powerful predictor in patients with premature ischemic heart disease.

Diastolic blood pressure also had prognostic value in predicting repeat revascularization. The association between blood pressure and cardiovascular outcome in high-risk patients has been debated. A recent study reported that in patients with diabetes mellitus and elevated risk, low DBP predicted a persistent risk of myocardial injury and even myocardial infarction, and that a high level of high-sensitivity TnT showed a U-shaped relationship with DBP (Bergmark et al., 2018). Our study confirmed this U-shaped relationship. We cannot confirm a causal link between DBP and elevated risk of an adverse outcome; however, we strongly demonstrated the association between these factors, which may be attributed to underlying comorbidities or to intensive blood-pressure-lowering therapy.

Our study has several limitations. First, this was a single-center retrospective study; prospective studies with larger study populations are needed. Second, we did not evaluate the plaque components during the procedures. Future studies evaluating the effect of plaque features on the prognosis of premature ACS patients are necessary. In our present study, we focus on the modifiable indicators that could predict the risks of revascularization since the intracoronary imaging is invasive and costs so much. Finally, we built a risk-prediction model, which needs to be verified in another cohort. Moreover, we observed differences in baseline characteristics in young patients with and without repeat PCI; the underlying mechanisms for these differences need to be clarified.

## Conclusions

In patients with premature ACS, HbA1C was a significant, independent predictor of repeat revascularization, with more predictive value than traditional lipid profile parameters. A routinely screening for disorders of glucose metabolism and a risk stratification of premature ACS patients was highly recommended.

## Supplemental Information

10.7717/peerj.6804/supp-1 Supplemental Information 1 Raw data.

## Additional Information and Declarations

### Data Availability

The following information was supplied regarding data availability: The raw data are available in File S1. The raw data shows basic characteristics of all the subjects. All the statistical analysis were based on the data.
